# INtegration of DEPression Treatment into HIV Care in Uganda (INDEPTH-Uganda): study protocol for a randomized controlled trial

**DOI:** 10.1186/1745-6215-15-248

**Published:** 2014-06-25

**Authors:** Glenn J Wagner, Victoria Ngo, Peter Glick, Ekwaro A Obuku, Seggane Musisi, Dickens Akena

**Affiliations:** 1 RAND Corporation, Santa Monica, CA, USA; 2 Makerere University, College of Health Sciences, Kampala, Uganda; 3 Joint Clinical Research Centre, Kampala, Uganda; 4 Department of Epidemiology and Population Health, London School of Hygiene and Tropical Medicine, University of London, London, UK; 5 Department of Psychiatry, Makerere University, Kampala, Uganda

**Keywords:** Depression, Task-shifting, HIV, Uganda

## Abstract

**Background:**

Despite 10 to% of persons living with HIV in sub-Saharan Africa having clinical depression, and the consequences of depression for key public health outcomes (HIV treatment adherence and condom use), depression treatment is rarely integrated into HIV care programs. Task-shifting, protocolized approaches to depression care have been used to overcome severe shortages of mental health specialists in developing countries, but not in sub-Saharan Africa and not with HIV clients. The aims of this trial are to evaluate the implementation outcomes and cost-effectiveness of a task-shifting, protocolized model of antidepressant care for HIV clinics in Uganda.

**Methods/Design:**

INDEPTH-Uganda is a cluster randomized controlled trial that compares two task-shifting models of depression care - a protocolized model versus a model that relies on the clinical acumen of trained providers to provide depression care in ten public health HIV clinics in Uganda. In addition to data abstracted from routine data collection mechanisms and supervision logs, survey data will be collected from patient and provider longitudinal cohorts; at each site, a random sample of 150 medically stable patients who are depressed according to the PHQ-2 screening will be followed for 12 months, and providers involved in depression care implementation will be followed over 24 months. These data will be used to assess whether the two models differ on implementation outcomes (proportion screened, diagnosed, treated; provider fidelity to model of care), provider adoption of treatment care knowledge and practices, and depression alleviation. A cost-effectiveness analysis will be conducted to compare the relative use of resources by each model.

**Discussion:**

If effective and resource-efficient, the task-shifting, protocolized model will provide an approach to building the capacity for sustainable integration of depression treatment in HIV care settings across sub-Saharan Africa and improving key public health outcomes.

**Trial registration:**

INDEPTH-Uganda has been registered with the National Institutes of Health sponsored clinical trials registry (3 February 2013) and has been assigned the identifier NCT02056106.

## Background

Over 25 million people living with HIV (PLWHIV) reside in sub-Saharan Africa (SSA), including over 1 million in Uganda [1]. Scaled-up access to HIV antiretroviral therapy (ART) has led to nearly 10 million in SSA and just under 300,000 Ugandans receiving treatment by the end of 2012 [1], resulting in dramatic declines in mortality [2,3] and HIV becoming a chronic, manageable disease [4]. ART also contributes to HIV prevention by reducing infectiousness [5,6], and being associated with increased condom use [7,8]. However, depression has emerged as a major threat to the success and benefits of ART, as it impedes ART adherence [9-11] and condom use [12-14], and has been associated with a greater likelihood of mortality [15-17] and worse immunologic and virologic response to treatment [18-21]. Clinical depression, as diagnosed by structured clinical interviews, generally ranges from 10 to 20% among PLWHIV in SSA [22-24], while an additional 20 to 30% have elevated depressive symptoms [23-27]. A wide range of interventions are effective in treating depression in PLWHIV [28], including antidepressants [29,30], and depression treatment improves ART use, adherence and outcomes [31-34]. Yet despite the prevalence of depression and its consequences for the fight against HIV, depression treatment is rarely integrated into HIV care programs in SSA [35].

A severe shortage of mental health professionals is a key barrier to depression care in Uganda and the larger region [36]. There are just over thirty psychiatrists in Uganda or one for every one million citizens, and most are in or near the capital city Kampala. Given the lack of psychiatrists, it is generally left to primary care providers to diagnose and treat depression. However, whether it is due to lack of psychiatric training, lack of appreciation of the value of mental health care, or lack of time and resources, providers have shown a reluctance to engage in mental health treatment in Uganda, even though antidepressants are on the national formulary and free of charge. Perhaps in part because depressed patients often present first with somatic symptoms, providers prefer to first rule out possible physical and medical causes rather than evaluating mental illness [36]. Often it is only when mental illness is so advanced that it is causing obvious disruption and dysfunction, do providers consider mental illness and then attempt to refer out to psychiatric specialists [36]. Even if depression care training were provided and primary care providers had a greater appreciation for mental health care, most HIV clinics in Uganda have only 1 clinical or medical officer for seeing 50 to 100 patients daily; so the concern is that many depressed patients would still go undetected, and bottlenecks of patients waiting for providers would worsen.

Understanding how to effectively implement evidence-based treatment in HIV settings, where need is high and mental health capacity is virtually non-existent is essential. With the emphasis on developing feasible, sustainable models of health care delivery in resource-poor settings, task-shifting models have presented a solution to scarce highly trained health professionals [37,38]. Task-shifting involves training lower cadres to take on many of the duties traditionally performed by more highly trained providers who are less available. Task-shifting, nurse-driven care has been shown to result in equivalent levels of quality of care in the context of HIV care and ART scale-up in SSA [39,40]. As a result, task-shifting is an approach widely used to facilitate HIV care in Uganda, with nurses often assuming the role of primary care provider, and a clinical or medical officer being present for oversight and to handle more complicated cases. This task-shifting approach has not been adapted for management of depression treatment for HIV patients, but ‘collaborative care’ models of depression treatment have been implemented with non-HIV patients in the US [41-44] as well as resource constrained settings [45-47]. These models use a team-based approach, typically consisting of a depression care manager (often a nurse), primary care provider and supervising psychiatric specialist in medical settings, and a structured, algorithm-based protocol that enables the care manager to take over many of the responsibilities of antidepressant therapy management. Controlled studies have demonstrated the effectiveness of collaborative care models [48-51]; furthermore, algorithm-based, protocol-driven care has been associated with better quality of care and treatment outcomes [43,46,48,49], as the structured approach results in greater likelihood of adequate treatment dosage and duration [52].

INDEPTH (INtegration of DEPression Treatment in HIV care)*-*Uganda is a cluster randomized trial that attempts to identify an effective, resource-efficient model for integrating depression treatment into HIV care in low resources settings such as Uganda. In the HIV clinics of ten health care facilities, the trial is comparing two active task-shifting implementation models of depression care: a *protocolized model* in which care is provided largely by trained nurses who act as depression care managers, and a model that relies on the *clinical acumen* of trained primary care providers (most, but not all, of whom are also nurses). An alternative approach to the use of a structured protocol to guide depression care by non-mental health professionals, the clinical acumen model (like the protocolized model) takes an active approach to depression care by integrating a brief routine depression screening process for all patients at each clinic visit, but what the primary care provider does with this screening information is left to their discretion as opposed to following a structured protocol. So, both of these models are active task-shifting models of depression care, as the clinical acumen model goes beyond current usual care, which relies solely on primary care provider to assess and treat, or refer to external specialists - which has resulted in depression being severely under-diagnosed and treated.

According to the RE-AIM (Reach, Effectiveness, Adoption, Implementation, Maintenance) implementation framework, to have an impact on health at the population level, an intervention must be *adopted* by providers, *reach* a large proportion of the patient population, be *implemented* with fidelity, *effectively* improve outcomes, and be *maintained* post study [53,54]. Accordingly, the objectives of INDEPTH-Uganda are to compare the two task-shifting models on (1) reach (screening and follow-up rates), adoption (treatment uptake), fidelity (quality of depression care implementation by the providers), and effectiveness (alleviation of depression symptoms) implementation parameters, and (2) cost-effectiveness. A secondary objective is to assess the impact of depression treatment on key economic (work) and public health (ART adherence, condom use) outcomes. Our hypothesis is that the protocolized model will result in better implementation of depression care, better overall alleviation of depression, and better cost-effectiveness, compared to clinical acumen. If effective and resource-efficient, the protocolized model will provide an approach to building the capacity for sustainable provision of depression treatment across SSA and improving key public health outcomes of HIV care.

## Methods

### Study design

INDEPTH-Uganda is a comparative trial that compares two active task-shifting implementation models for integrating antidepressant treatment into HIV care within ten health care facilities in Uganda. Using a cluster randomization, five clinics are assigned to implement a protocolized model, and five others rely on the clinical acumen of trained providers. One of the investigators (EO) will generate a random list of numbers using computer software which will be printed on opaque pieces of paper that will be folded and drawn by clinic representatives in a meeting in which representatives from all clinics are present. To ensure the two study arms are balanced on size of clientele (small clinics serve 300 to 1,000 clients, while larger clinics serve 1,500 to 3,000 clients), which could influence depression care processes, randomization will be conducted within pairs of clinics that were matched on this variable. The models will be implemented over 24 months starting in spring 2013. To evaluate the models, data will be collected from documentation mechanisms integrated into routine care, and from random samples of 150 patients enrolled at each site (total n = 1,500) that screen positive for possible depression and are followed for 12 months. We will compare the two models on uptake of antidepressant treatment, and change in depression (treatment response), as well as other quality of care and implementation outcomes as outlined by the RE-AIM implementation framework. This cohort will also be used to examine the relationship between change in depression and key economic and public health outcomes (for example, work status, condom use, HIV treatment adherence). A cohort of providers from each site will also be enrolled and followed over the 24-month implementation to examine change in knowledge, attitudes and practices regarding depression care and perceived impact of the implementation on clients, providers and clinic operations. Patient and provider survey data will be collected by trained research coordinators who will not be blinded to the treatment condition, nor will the patient participants. All patient and provider participants will provide written informed consent prior to enrollment. Lastly, a cost-effectiveness analysis will be conducted to examine the relative cost and resources needed to implement each model. The study protocol has been approved by Institutional Review Boards at RAND Corporation (protocol # 2012-0115), Mildmay Uganda, and the Uganda National Council for Science and Technology.

The success of the study depends on engagement with, and commitment from, the leadership and staff of the health care facilities, district level administration, and the Ministry of Health. This is critical to the program being feasible, responsive to the local context of the clinics, and thus also sustainable once the project has ended. A kick-off meeting with all key stakeholders will be held to review the care models and elicit feedback on logistical implementation, as well as to secure buy-in and partnership in the study. A Core Planning Group, with representatives from all cadres involved in the model (expert clients, nurses, clinical/medical officers), will be convened on a quarterly basis, as well as consultation with district health administration leadership, and Dr. Sheila Ndyanabangi (mental health point person in the Ministry of Health). With the goal of establishing a model of depression care that is sustainable and scalable, the focus of this group is to engage in an iterative process for planning and coordinating implementation, trouble-shooting any challenges that arise, and deciding on necessary modifications or refinements to the models.

### Study setting

The study is being conducted in collaboration with Mildmay Uganda, a non-government organization that provides holistic outpatient HIV care at its own clinics, trains health care workers throughout Uganda and the region, and provides technical assistance in HIV care to health care facilities across Uganda. Of the ten health care facilities participating in the study, eight are run by the Ministry of Health and two are private, faith-based, not-for-profit health care facilities; two are district hospitals and the others are designated as health centre III or IV facilities by the Uganda Ministry of Health, and are located in the districts of Mpigi, Mityana, Luweero, and Wakiso. Each facility is a hospital that operates an HIV clinic on specific designated days of the week, and it is in these clinics that depression care is being integrated as part of this study. The 6 larger clinics operate 2 to 3 days per week and generally have 1 clinical or medical officer and 3 to 5 nurses to provide primary HIV care to a clientele ranging from 1,500 to 3,000 clients and 80 to 120 patients are seen each clinic day. The 4 smaller clinics operate 1 day per week and are manned by 1 clinical or medical officer and 2 to 3 nurses; client base ranges from 350 to 1,000, with 40 to 60 patients seen each day.

Consistent with task-shifting approaches becoming increasingly common in the context of ART scale-up across the region, at all sites nurses serve as primary care providers (along with the clinical/medical officers), and manage the prescription and monitoring of ART and other common HIV medications; more complex conditions or complications that arise are typically managed by the clinical/medical officer. All clinics have expert clients (volunteer experienced HIV clients who display exemplary HIV care adherence and are trained to provide peer support and assist in lower level tasks) and village health team (VHT) workers (community lay volunteers) who volunteer to take on tasks such as triage assessments, filing and retrieving charts, and keeping the clinic clean. No psychiatric or depression care services were being provided at these clinics prior to the study; clients who developed significant psychiatric symptoms are referred to the nearest district or regional hospital for care.

## Task-shifting depression care models

### Protocolized model

Drawing from collaborative care models of depression treatment such as Partners in Care, MANAS and STAR*D [42,44,46,47], the protocolized model implements an algorithm-based, nurse-driven approach to administering depression diagnosis and antidepressant therapy. While other collaborative care models incorporate psychotherapy and reserve antidepressants for severe depression, our model focuses solely on the use of antidepressants. We believe that use of medication requires relatively less trained resources and personnel time than would be needed to add a form of psychotherapy, resulting in a more reliable and scalable treatment for resource constrained settings. The components of the protocolized model are outlined below and summarized (in comparison with the clinical acumen model) in Table 1.

**Table 1:** Components of the protocolized and clinical acumen depression care models

	**Protocolized model**	**Clinical acumen**
**Training**	•One day training for all involved cadre	•One day training for all involved cadre
	•On-site training and oversight by study staff during first four weeks of implementation	•On-site training and oversight by study staff during first four weeks of implementation
	•Periodic training schedule to train new staff that emerge (typically site specific)	•Periodic training schedule to train new staff that emerge (typically site specific)
**STEP 1:** Routine Screening	•All adults screened with 2-item PHQ-2 at each clinic visit	•All adults screened with 2-item PHQ-2 at each clinic visit
	•Performed at triage station (Expert-clients/VHTs)	•Performed at triage station (Expert-clients/VHTs)
	•PHQ-2 > 2 signifies depression, continue to Step 2	•PHQ-2 > 2 signifies depression, continue to Step 2
	Documentation: Clinic’s triage book	Documentation: Clinic’s triage book
**STEP 2:** Diagnosis	•Is patient’s treatment status (either ART or OI treatment) in flux (about to start or newly started)? (Nurse) YES = not medically stable, so wait and monitor NO = medically stable, administer PHQ-9	•PHQ-2 score is relayed to prescribing clinician
		•Prescribing clinician uses their judgment to determine whether to further evaluate depression, rather than a protocol
	•Administer PHQ-9 (Nurse)	•However, prescribing clinicians are trained to follow-up positive PHQ-2 screens with:
	•PHQ-9 > 9 signifies clinical depression	1) Assessment of medical stability
	•Consider MINI criteria to determine diagnosis	2) PHQ-9
	•If meets criteria for diagnosis, continue to Step 3	3) Consider MINI criteria
	Documentation: Patient’s medical chart	Documentation: Patient’s medical chart
**STEP 3:** Prescribe treatment	•Provide psychoeducation to client about depression and what to expect from antidepressant treatment (Nurse)	•Provide psychoeducation to client about depression and what to expect from antidepressant treatment (Prescribing Clinician)
	•Select most appropriate antidepressant	•Select most appropriate antidepressant
	•Prescribe antidepressant	•Prescribe antidepressant
	•Schedule follow-up visit two weeks later	•Schedule follow-up visit two weeks later
	Documentation: Depression Treatment Registry	Documentation: Depression Treatment Registry
**STEP 4:** Monitor treatment	•Follow-up at Week 2 and then monthly until responding, then scheduled with routine clinic visit	•Follow-up at Week 2 and then monthly until responding, then scheduled with routine clinic visit
	•At each follow-up, assess side effects, symptoms and need for medication or dose change (Nurse)	•At each follow-up, assess side effects, symptoms and need for medication or dose change (Prescribing Clinician)
	Documentation: Depression Treatment Registry	Documentation: Depression Treatment Registry
**STEP 5:** Stop treatment	•Stop treatment once patient responding for six months (unless patient has had 2+ prior episodes, then continue for two years)	•Stop treatment once patient responding for six months (unless patient has had 2+ prior episodes, then continue for two years)
	•Taper down dosage using dosing protocol	•Taper down dosage using dosing protocol
	Documentation: Depression Treatment Registry	Documentation: Depression Treatment Registry
Supervision	•Psychiatrist assigned to each clinic	•Psychiatrist assigned to each clinic
	•Supervision visits are done monthly	•Supervision visits are done monthly

Legend: ART, antiretroviral therapy; OI, opportunistic infection; MINI, Mini Neuropsychiatric Interview; PHQ, Patient Health Questionnaire; VHT, village health team.

#### Routine depression screening

**Figure 1:**
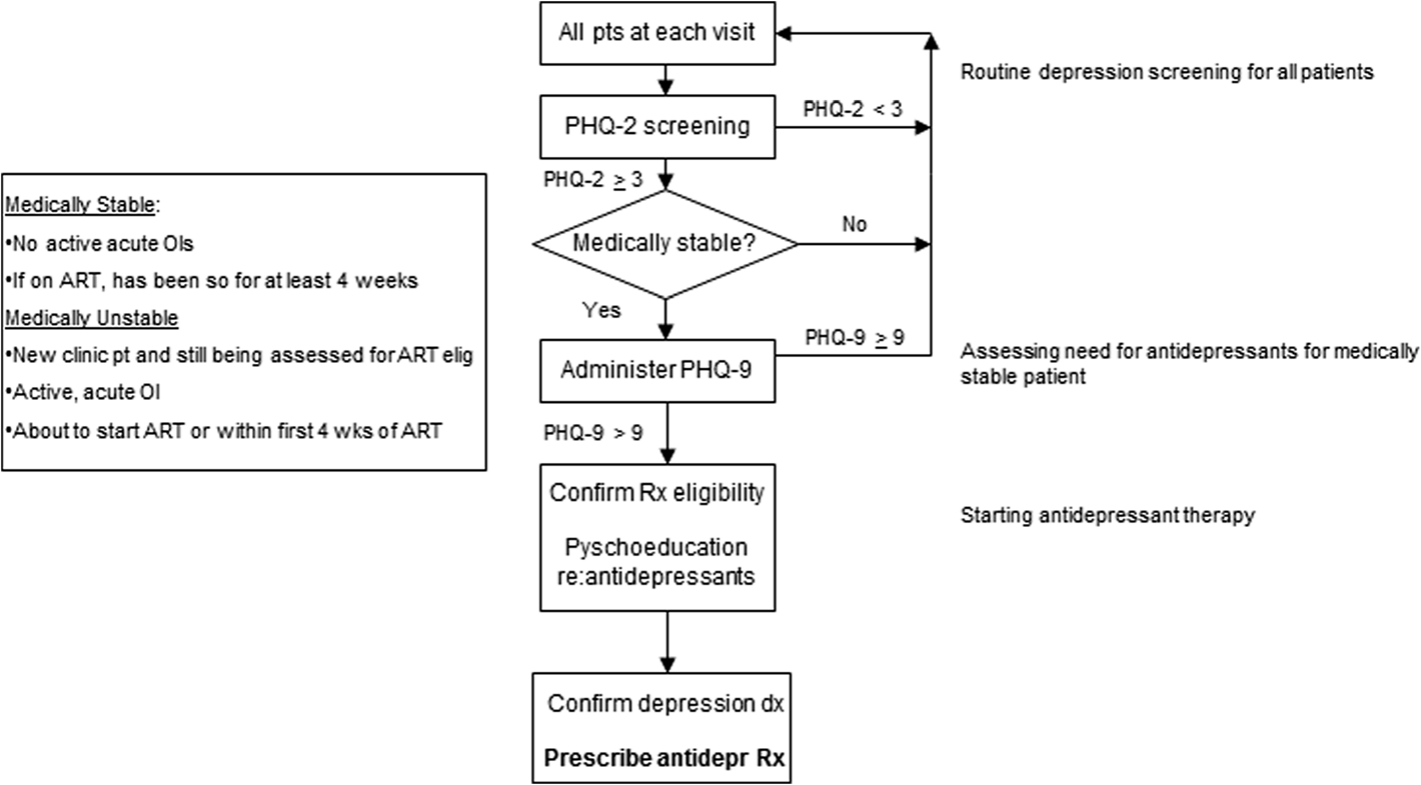
**Depression and treatment eligibility assessment protocol.** PHQ = Patient Health Questionnaire. OI = opportunistic infections. Rx = treatment. Dx = diagnosis. ART = antiretroviral therapy.

All adult clinic patients will be screened for depression at each clinic visit using the first two items of the Patient Health Questionnaire (PHQ-2) [55], administered at the triage station (along with measurements of body weight, blood pressure, pulse and elicitation of presenting health problems) by expert clients or VHT workers. The PHQ-2 assesses depressed mood and loss of interest; patients who screen positive for depression (3+ scores; range: 0 to 6) will be referred to the nurse in the protocolized model for further depression evaluation if medically stable; in the clinical acumen model, PHQ-2 data are relayed to the primary care provider. Medical stability is defined as not about to start (or recently started) ART or treatment for an acute opportunistic infection (see Figure 1). Further evaluation is deferred for medically unstable patients until their condition and treatment is stable as mood may improve once they are medically stable, and starting antidepressants simultaneously with other medical treatments would complicate side effect and response evaluation. However, depressed patients who are medically unstable will be evaluated for suicide risk and the need for immediate treatment.

#### Depression diagnosis

Further evaluation of patients who screen positive for depression and are medically stable will be conducted using the full nine-item Patient Health Questionnaire (PHQ-9) [55]. Patients who score greater than 9 on the PHQ-9 (range: 0 to 27), which has been shown to correspond highly with major depression (88% sensitivity and specificity) as determined by a diagnostic interview [55], will be further assessed using the criteria for major depression on the Mini Neuropsychiatric Interview (MINI; five symptoms scored as three on the PHQ-9, at least one of which is depressed mood or loss of interest) [56]. Providers are trained to further assess antidepressant eligibility using the MINI screening items for bipolar disorder, psychosis and substance abuse, as well as medical contraindications (for example, pregnancy or breast feeding, seizure disorder); for patients with these conditions, the supervising psychiatrist will be consulted to determine appropriate treatment.

#### Prescription of antidepressants

If eligibility for antidepressant therapy is confirmed, the provider (nurse in the protocolized model and primary care provider in the clinical acumen model) will educate the patient about depression as a disease, and antidepressant treatment*,* including: prognosis, the goal of symptom remission, that treatment often takes two to three weeks to have an effect, and that side effects are generally minor and temporary. Helping the patient to understand the treatment is intended to encourage adherence to treatment. The patient will then receive a prescription and supply of either fluoxetine or imipramine prior to departing the clinic, which the provider will select based on the patient’s presenting symptoms and psychiatric history; fluoxetine will be prescribed unless the patient presents with insomnia, sexual dysfunction or history of bipolar disorder. Fluoxetine has been shown to have some drug interactions with HIV protease inhibitors [57], but protease inhibitors are rarely used in Uganda.

#### Monitoring of treatment response and side effects

After treatment prescription, the patient will return to see the prescribing provider two weeks later for monitoring of side effects, treatment response and any need for change in dosage or medication. The visit schedule will then become monthly until the patient has been in remission (defined as PHQ-9 < 5 and tolerating any side effects) for one month, at which time the visit schedule will be modified to match the patient’s HIV care visit schedule (typically every two to three months), but no less than every three months; if the patient experiences a relapse, visits will return to monthly until in remission. At each visit, the provider will assess depressive symptoms (using the PHQ-9), presence of side effects and strategies for managing such symptoms, as well as enquire about medication adherence. Psychoeducation regarding depression treatment will continue and be emphasized as needed. A Depression Treatment Registry will been instituted at each site in which the above listed parameters are recorded for each visit, as well as medication and dosage prescribed. These data will be reviewed during supervision and used for reference in future follow-up visits, as well as for tracking fidelity to the treatment protocol.

**Figure 2:**
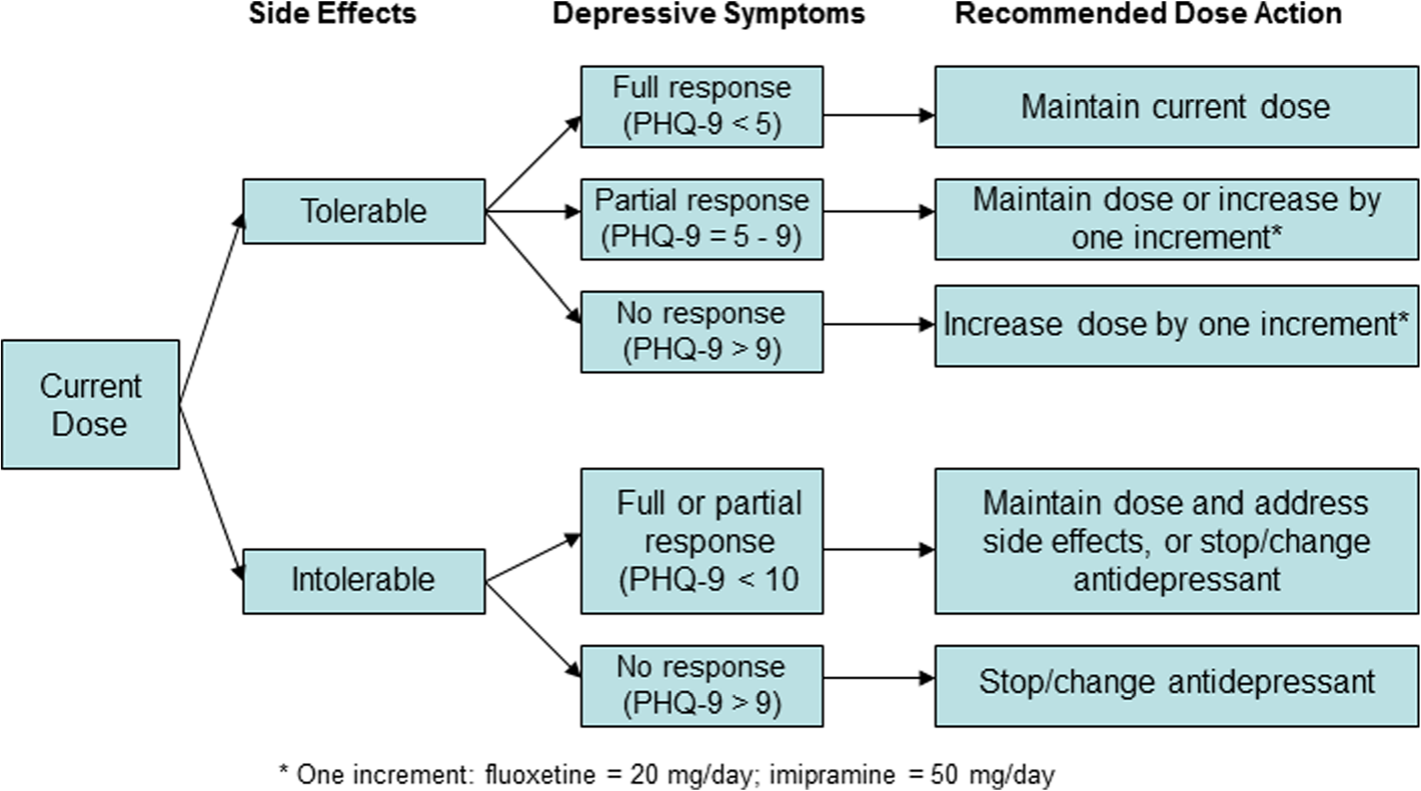
**Algorithm for dose or medication changes.** PHQ = Patient Health Questionnaire.

Starting daily dose is 20 mg for fluoxetine and 50 mg for imipramine; imipramine will increase to 75 mg after one week. Patients remain on this low dose until their second follow-up visit (Week 6), at which time the determination of the need for a change in dose or medication will be considered based on measures of depressive symptoms and side effects (see Figure 2). *For patients tolerating any side effects*: dose will remain the same if they are fully responsive to treatment; for partial responders, the provider will decide whether to maintain the dose or increase by one increment (add 20 mg of fluoxetine or 25 mg of imipramine); nonresponders will have their dose increased by one increment. *For patients not tolerating side effects*: if a full or partial response to treatment, the current dose may be maintained and side effects addressed (with pharmacologic or other types of strategies) or the provider may reduce the dose or change the antidepressant; for nonresponders, the antidepressant will be changed.

This algorithm-based treatment decision process is repeated at each monthly visit until the patient is fully responding and in remission - the medication dose will then remain the same (unless subsequent changes are needed due to side effects or relapse). Maximum dose is 80 mg/day for fluoxetine and 300 mg/day for imipramine. If patients do not respond to or tolerate fluoxetine or imipramine, amitriptyline is an option; alternatively, a medication that is being partially responded to may be continued in the hope that response will improve over time. Concomitant medications for associated symptoms such as sleep disturbance, anxiety or agitation, or side effects (for example, sexual dysfunction), may be used and will be documented.

#### Treatment discontinuation

Once the patient has been in remission for at least six months, treatment will be discontinued unless the patient has a history of multiple episodes of major depression, in which case treatment will be maintained for two years [58]. If on treatment at end of study, treatment will be maintained as part of usual care. Drug supply is purchased by the study to ensure no drug stock outs, but both medications are available free of charge to the clinics through the Ministry of Health.

### Clinical acumen model

Although psychiatric treatment is generally not currently available in HIV clinics (nor general primary care clinics) in Uganda, clinics that are looking to increase their provision of depression treatment are likely to follow the model used in many other parts of the world, which is to train primary care providers to identify and treat depression. Similarly, the clinical acumen model of depression care relies on the discretion of primary care providers who receive depression care training to provide depression diagnosis and treatment as deemed warranted. To facilitate this process, like the protocolized model, the clinical acumen arm also includes routine screening of all adult clients and monthly on-site supervision from study psychiatrists. However, this model relies on the clinical judgment of the primary care provider to decide whether to further evaluate and treat patients who screen positive for possible depression, as opposed to a structured protocol (see Table 1 for an outline of the model and how it compares to the protocolized model). Another difference between the models is that depression care is managed entirely by nurses (with oversight from the clinical/medical officer in charge) in the protocolized model, whereas in the clinical acumen model all primary care providers (who are comprised of nurses and clinical/medical officers) are expected to provide depression care.

### Training, supervision and monitoring

Training and ongoing supervision are critical for ensuring quality of care and fidelity to the depression care models, and to provide technical and emotional support to the providers. With management of antidepressant treatment being new to the clinic staff, the supervision is particularly important and will help to increase the confidence of the providers in their ability to provide high quality care. Supervision is also an opportunity for the providers to refresh and upgrade their new skills in an ongoing forum that reinforces the value of the investment they have made in the training and the integration of depression care into their HIV care practice. Furthermore, working with patients who are depressed can be stressful and emotionally draining, so the support from supervision can help prevent burnout and job turnover. The training and supervision provided to the clinic staff in both arms of the study is very similar; the only differences are that the providers in the clinical acumen arm will not be trained to follow the parameters of a structured protocol when further assessing patients who have screened positive for possible depression.

#### Start-up training

Training will start with the study’s lead investigators and psychiatrists conducting an intensive one-day training workshop with the clinic staff (expert clients/VHTs, nurses, clinical/medical officers) to train them on the structure and goals of the study, and each component of the treatment model. One workshop will be held for the clinics in the protocolized arm, and a separate workshop for those in the clinical acumen arm. All nurses and clinical/medical officers at each site will be trained so that duties can be evenly spread, to build overall capacity for depression care, and to mitigate against the effects of occasional staff transfers. Training will consist of didactic instruction, interactive role-playing, and small breakout group discussions. Following the workshop, tools to facilitate implementation of the model (laminated copies of the PHQ-2 for the triage station, and PHQ-9/MINI for each nurse/prescribing provider, Depression Treatment Registry, psychoeducation posters and flipcharts) will be delivered to each site, and supervising psychiatrists will be on-site one day a week for four to six weeks (until both the supervisor and nurse are comfortable with the nurse’s competency in implementing the protocol) to provide training and mentorship (for example, sitting in on and co-conducting assessments), after which ongoing on-site supervision will become monthly.

#### Ongoing supervision

Supervision will be conducted in one-on-one sessions between the site supervisor and each nurse (protocolized arm) and primary care provider (clinical acumen arm), as well as group meetings with all clinic staff involved with implementation of depression care at the site. Furthermore, the supervisor will be constantly available, day or night, for emergency or suicide crisis consultations. During individual supervision, the providers will present new treatment cases since last supervision and problematic or nonresponding cases, allowing the supervisor to discuss the patient’s presenting symptoms and the treatment plan for the patient, including side effect management and dose change recommendations. Clinical notes from the Depression Treatment Registry will be used in the case reviews. Goals of treatment for the individual patient will be discussed and recorded for review at subsequent supervision sessions. The group meetings will provide an opportunity for the clinic staff to work as a team to trouble-shoot any challenges that arise, share experiences and provide peer support to each other in managing depressed patients, which can be emotionally taxing as well as fulfilling.

#### Monitoring fidelity

Ongoing supervision and review of cases is our primary mechanism for monitoring the fidelity of the implementation of the treatment models. At each monthly supervision supervisors review the charts of all patients prescribed antidepressants within the past month, as well as ten randomly selected charts of patients receiving ongoing antidepressant treatment monitoring. The charts will be reviewed for whether diagnosis, symptoms and side effect assessment, and dosing were appropriately performed, and if the patient returned for follow-up visits. Results will be aggregated and monitored on a monthly basis, as well as used to inform the target areas for supervision.

### Evaluation methods for comparing the depression care models

To evaluate the two task-shifting models, we will compare the implementation, effectiveness, and cost-effectiveness of these models over a 24-month period. Using the RE-AIM framework, we will evaluate the *reach* of depression screening and treatment, *adoption* of depression treatment knowledge and intervention by providers, *quality of implementation* (fidelity to depression care model), and *effectiveness* of depression treatment on patient outcomes. As outlined in Table 2, these implementation domains and corresponding outcomes will be assessed from data triangulated from these data sources: (1) data abstracted from data collection mechanisms integrated into routine care; (2) monthly logs summarizing chart abstracted data completed by the supervising psychiatrists; (3) survey data from a longitudinal cohort of randomly selected patients at each site who screen positive for potential depression (PHQ-9 > 2); and (4) survey data from longitudinal cohort of providers involved in provision of depression care at each site. Survey data from the patient cohort will also be used to assess how change in depression is related to key economic and public health outcomes. These data sources are described in more detail below.

**Table 2:** Implementation evaluation domains, outcomes measures, and data sources

**RE-AIM Domain**	**Outcome measure**	**Sample**	**Data source**
**Reach**	% screened with PHQ-2	All adult clinic patients	Routine data abstraction (Triage Book)
	% screened positive (PHQ-2 > 2)	All adult clinic patients	Routine data abstraction (Triage Book)
			Accuracy of positive screens assessed with PHQ survey data from patient longitudinal cohort
	% positive screens who receive further evaluation (PHQ-9/MINI)	Patient cohort	Routine data abstraction (clinic charts)
	% positive screens who are depressed (PHQ-9 > 9)	Patient cohort	Routine data abstraction (clinic charts)
			Accuracy of provider PHQ-9 assessed with PHQ survey data
	% depressed who receive antidepressants (treatment uptake)	Patient cohort subset (baseline PHQ-9 > 9)	Routine data abstraction (Depression Treatment Registry)
**Adoption**	Provider knowledge, attitudes and practices regarding depression care	Provider cohort	Provider survey data
**Implementation quality** (fidelity)	Initial prescription: Appropriate diagnosis, treatment prescription (medication and dose)	Clinic level data	Supervision log data (aggregate data from monthly chart reviews of newly prescribed patients)
	Treatment management: Appropriate assessment of symptoms, side effects, and prescription (medication and dose)	Clinic level data	Supervision log data (aggregate data from monthly chart reviews of ongoing treatment patients)
**Effectiveness**	Alleviation of depression	Patient cohort subset (baseline PHQ-9 > 9)	Patient PHQ-9 survey data
	Improvement in condom use, work status and ART adherence	Patient cohort subset (baseline PHQ-9 > 9)	Patient survey data

Legend: PHQ, Patient Health Questionnaire.

Primary outcomes are (1) proportion of depressed (defined as PHQ-9 > 9 at baseline) patient cohort participants who receive antidepressant treatment; and (2) depression alleviation (reduction in PHQ-9 score). Secondary outcomes include (1) proportion of depressed patient cohort participants who receive further depression evaluation; (2) consistent condom use (measures with self-report); (3) HIV treatment adherence (measured with self-report).

#### Routine data abstraction

We will assess the implementation of screening (% of adults screened; % screened who screen positive) by abstracting data from the Triage Book, which the triage station at each site uses to list the clients who attend each day of the clinic, their vital signs, and PHQ-2 score. PHQ-9 and MINI administration data will be recorded in the patient’s clinic chart. Depression Treatment Registry books will be installed at each site for providers to document clients who have started antidepressant therapy and their depression diagnosis, and records of each follow-up visit that include PHQ-9 score, presence of side effects and medication and dose prescribed.

#### Supervision log data

From the chart reviews that the supervisors conduct each month at each site, monthly aggregate scores are recorded for newly prescribed patients (number with correct diagnosis, correct prescribed antidepressant, and correct prescribed dosage) and patients in ongoing treatment (number correctly assessed for depressive symptoms and side effects; number correctly dosed; and number who returned for scheduled follow-up visit in past month).

#### Longitudinal client cohort

Over the course of 12 months at each site, research coordinators (Masters level graduates with social science training) will recruit random samples of 150 patients (total cohort = 1,500 patients) who the triage personnel screen as positive for potential depression (PHQ-2 > 2), who are confirmed to be positive in the research coordinator’s own PHQ-2 administration, and who are determined to be medically stable. This sample will be followed for 12 months with the baseline assessment administered at the same visit in which consent is obtained followed by assessments at Months 6 and 12. The surveys will be administered by the research coordinator using computer assisted personal interview technology, and will include the PHQ-9 (to independently assess the presence of depression) as well as measures of demographic and background characteristics, work activity, clinical appointment and ART adherence, quality of life, sexual behavior and measures of psychosocial functioning. All measures have been used successfully in our prior research in Uganda and have been translated into Luganda, the primary native language used in the study setting. Participants will receive 10,000 Ush (approximately US$4 ) for each assessment.

#### Longitudinal provider cohort

A brief survey of knowledge, attitudes and practices regarding depression care and its influence on patients and clinic operations will be administered to a cohort of providers actively involved in implementing depression care at each site. The measures will include perceived competency in providing depression care, quality of depression treatment provided, effects of the treatment model on clinic functioning, provider burden and patient flow, and impact of depression treatment on the patient’s quality of life. Surveys will be administered prior to the start of implementation and then at Months 6, 12 and 24 by the research coordinators using computer-assisted personal interview software.

### Data analysis plan

#### Power analysis

We calculated the size of effects that our sample would be powered to detect with regard to treatment uptake and depression alleviation using a range of intracluster correlation coefficient (ICC) values. Data from developing countries indicate low correlations of health outcome variables within primary care settings, including .03 for depression in a collaborative care trial in India [46], and .16 for treatment process measures from a study conducted in eight Latin American countries [59]. With 150 patients per site and assuming 30% antidepressant uptake in the clinical acumen arm and ICC = .16, we will be able to detect an increase of 28% in treatment uptake in the protocolized arm (α = .05, 80% power; 1-sided test). For measuring change in depression (PHQ-9) in an intention to treat (ITT) analysis using the whole sample, with ICC = .025, SD = 3.9 (from past research), and 10% attrition, we will have power to detect a 1.26 difference on the PHQ-9 (α = .05, 80% power; 2-sided test); for ICC = .01 and .05, detectable differences are .92 and 1.71. These correspond to medium effect sizes (Cohen’s *d*) of .33, .40 and .49.

#### Statistical analysis

The analyses must account for clustering of the data, unless the only comparisons are of cluster means or proportions, using clinic as the unit of analysis. For most outcomes we will perform such an analysis first, using standard *t*-tests or, where the observations per clinic vary, *t*-tests weighted by cluster-size [60]. However, relative to individual level analysis this approach is limited by low power (low number of clusters); therefore, we will use regression methods on individual level data for most analyses and since the number of clusters is too small to reliably estimate the ICC [61], we will explore the sensitivity of significance levels and conclusions to a range of plausible ICC values for the outcomes,

We will use logistic regressions to compare the proportion of depressed patients (according to the survey PHQ-9) receiving depression treatment in the two arms. The treatment uptake outcome will be estimated using covariates to adjust for baseline characteristics such as age, sex, and physical health. We will add interactions of these covariates with study arm to assess which types of patients are likely to be treated in the protocolized versus clinical acumen arms. A similar approach will be used for other implementation outcomes such as proportion of positive screens who receive further evaluation with the PHQ-9. The effect of each model on depression alleviation (change in PHQ-9) will be examined using an ITT approach and the whole sample. We will use a repeated measures, mixed-model approach allowing for correlation of errors over time for an individual. The model has the following form:

$$\begin{array}{ll} {\mathrm{Depression}}_{\mathrm{it}}= & \alpha +\beta {\left(\mathrm{treatment}\;\mathrm{model}\right)}_{i}+\gamma \left(t\right)\\ & +\;\delta {\left(\mathrm{treatment}\;\mathrm{model}\right)}_{i}\left(t\right)+\theta_{i}+\epsilon_{\mathrm{it}} \end{array}$$

Where *t* is time period, β controls for baseline differences between the two, γ is the trend in the outcome common to both groups, and δ is the interaction of treatment model with time and shows the intervention effect (protocolized model relative to the clinical acumen model). θ_i_ is an individual random effect and ϵ_it_ idiosyncratic period specific error. A vector of individual level covariates X_i_ will be added to estimate the effects of patient characteristics and interactions of X_i_ and study arm will be used to test for moderators: for example, do effects of the protocolized model vary by the patient’s physical health at baseline? The estimates will capture the average benefit for depressed clients, including those who do and do not receive antidepressant treatment.

### Cost-effectiveness analysis

This analysis will be performed in line with WHO CHOICE and CHEERS (Consolidated Health Economic Evaluation Reporting Standards) guidelines [62,63]. Using the patient cohort data we will compare the cost-effectiveness of the protocolized versus the clinical acumen models. Effectiveness will be measured in terms of Quality Adjusted Life Years (QALYs) derived from the Medical Outcomes Study-HIV (MOS-HIV) Study survey measures [64], calculated over all depressed clients, treated or not. Differences in effectiveness across the two arms will be driven in part by differences in the efficacy of treatment and depression alleviation. Sensitivity analyses will consider impacts on cost-effectiveness of variations in % screened, and % who receive diagnostic evaluation, as well as variation in cost and efficacy parameters. We will compute resource use and costs in international dollars using a societal perspective (that is including patient, clinic and Ministry of Health public costs). We will obtain overhead and annualized capital costs from the WHO-CHOICE database for Uganda; costs for drugs and human resources will be estimated from the Mildmay financial records and Uganda Public Service salary structure data.

## Discussion

With growing evidence of the damaging effects of depression on HIV prevention and care, and the relative absence of depression treatment in HIV care programs throughout SSA despite its high prevalence, INDEPTH-Uganda aims to identify an approach to integrating depression treatment into HIV care that is both effective and sustainable in resource constrained settings. With the severe shortage of psychiatric specialists in Uganda and SSA, and limited numbers of physicians to care for ever-growing numbers of PLWHIV in care, task-shifting models are essential, but establishing feasibility, efficacy and cost-effectiveness are critical for widespread implementation. Task-shifting, protocolized approaches to overcoming shortages of highly trained mental health professionals in the provision of depression treatment have been shown to be effective in resource-constrained settings and the US [41-51], but this may be the first study of such a model with PLWHIV, and in SSA. With active engagement and collaboration with key community stakeholders, policy makers and the clinic staff involved in implementation, we have sought to adapt a nurse-driven, protocolized model of depression care that is resource-efficient and not overly burdensome for already busy clinics, with the goal of being sustainable in the long term. If we demonstrate that this task-shifting, protocolized approach to depression treatment is feasible and cost-effective, it will establish a model that addresses the human resource challenges to building the capacity for sustainable depression treatment. Findings may have implications not only for PLWHIV in SSA, but also other developing regions, non-HIV populations, and possibly treatment of other diseases.

## Trial status

The trial is actively enrolling participants.

## Abbreviations

ART: antiretroviral therapy; CHEERS: Consolidated Health Economic Evaluation Reporting Standards; ICC: intraclass correlation coefficient; INDEPTH-Uganda: Integrating Depression Treatment into HIV Care in Uganda; ITT: intention to treat; MINI: Mini Neuropsychiatric Interview; MOS-HIV: Medical Outcomes Study-HIV; OI: opportunistic infection; PHQ: Patient Health Questionnaire; PLWHIV: people living with HIV; QALYs: Quality Adjusted Life Years; RE-AIM: Reach, Effectiveness, Adoption, Implementation, Maintenance; SSA: sub-Saharan Africa; VHT: village health team.

## Competing interests

The authors declare that they have no competing interests.

## Authors’ contributions

GW conceived of and led the design of the study, led the drafting of the manuscript, and gave final approval of the manuscript; VN contributed to the design of the study and the drafting of the manuscript, and gave final approval of the manuscript; PG led the design of the analysis plan and contributed to the drafting of the manuscript, and gave final approval of the manuscript; DA contributed to the design of the study and the drafting of the manuscript, and gave final approval of the manuscript; EO contributed to the design of the study and the drafting of the manuscript, and gave final approval of the manuscript; SM contributed to the design of the study and the drafting of the manuscript, and gave final approval of the manuscript.
